# Supplementary material for: Effect of Proton Pump Inhibitors on Risks of Upper and Lower Gastrointestinal Bleeding among Users of Low-Dose Aspirin: A Population-Based Observational Study
Source: J Clin Med. 2020 Mar 28;9(4):928. doi: 10.3390/jcm9040928 (PMC7230296; doi:10.3390/jcm9040928)
Supplement: Supplementary file 1 [file jcm-09-00928-s001.pdf]

Supplementary Table 1. Further characteristics of UGIB/LGIB cases and controls (all current users of low-dose aspirin).

	UGIB cases N=987		Controls N=2160		LGIB cases N=1428		Controls N=4100	
	n	%	n	%	n	%	n	%
**PCP visits**								
0–4	30	3.0	142	6.6	35	2.5	268	6.5
5–9	156	15.8	508	23.5	219	15.3	939	22.9
10–15	189	19.1	542	25.1	323	22.6	1128	27.5
15–19	205	20.8	399	18.5	304	21.3	764	18.6
≥20	407	41.2	569	26.3	547	38.3	1001	24.4
**Referrals**								
0–4	328	33.2	951	44.0	434	30.4	1816	44.3
5–9	294	29.8	654	30.3	458	32.1	1218	29.7
10–19	236	23.9	392	18.1	352	24.6	744	18.1
≥20	129	13.1	163	7.5	184	12.9	322	7.9
**Hospitalizations**								
None	645	65.3	1779	82.4	1015	71.1	3360	82.0
1	175	17.7	244	11.3	214	15.0	471	11.5
2	90	9.1	87	4.0	112	7.8	168	4.1
≥3	77	7.8	50	2.3	87	6.1	101	2.5
**Urban/rural**								
Urban	636	64.4	1394	64.5	921	64.5	2617	63.8
Town	124	12.6	244	11.3	178	12.5	526	12.8
Rural	58	5.9	137	6.3	116	8.1	280	6.8
Missing	169	17.1	385	17.8	213	14.9	677	16.5
**Hemodialysis**	6	0.6	2	0.1	4	0.3	5	0.1
**Hemodialysis extracorporeal**	3	0.3	0	0.0	3	0.2	0	0.0
**Hemodialysis peritoneal**	3	0.3	2	0.1	2	0.1	5	0.1
**eGFR (mL/min/1.73m^2)**								
0–14	9	0.9	6	0.3	2	0.1	8	0.2
15–29	35	3.5	35	1.6	31	2.2	81	2.0
30–44	109	11.0	171	7.9	104	7.3	267	6.5
45–59	189	19.1	484	22.4	255	17.9	771	18.8
≥60	555	56.2	1247	57.7	951	66.6	2638	64.3
Missing	90	9.1	217	10.0	85	6.0	335	8.2
**Other comorbidities**								
Ischemic stroke	110	11.1	182	8.4	134	9.4	320	7.8
TIA	97	9.8	211	9.8	136	9.5	304	7.4
Asthma	158	16.0	327	15.1	255	17.9	650	15.9
DVT	98	9.9	152	7.0	137	9.6	294	7.2
Anemia*	48	4.9	39	1.8	59	4.1	58	1.4
Atrial fibrillation	119	12.1	242	11.2	163	11.4	330	8.1
Heart failure	76	7.7	132	6.1	90	6.3	217	5.3
Gout	92	9.3	170	7.9	108	7.6	310	7.6
Pancreatic disease	11	1.1	12	0.6	11	0.8	25	0.6
Depression	246	24.9	437	20.2	396	27.7	863	21.0
Rheumatoid arthritis	47	4.8	62	2.9	52	3.6	148	3.6

*In the year before the index date.

Note: PCP visits, referrals and hospitalizations were ascertained in the year before the index date. Comorbidities were ascertained any time before the index date.

DVT, deep vein thrombosis; eGFR, estimated glomerular filtration rate; PCP, primary care practitioner; TIA, transient ischaemic attack

Supplementary Table 2. ORs (95% CIs) for the association between concomitant use of low-dose aspirin and a PPI and the risk of UGIB stratified by primary/secondary CVD prevention population.

	**UGIB N=987 n (%)**	**Controls N=2160 n (%)**	**OR (95 CI)***	**OR (95 CI)†**
Current use of low-dose aspirin and past use of a PPI (**reference group**)	223 (22.6)	423 (19.6)	1.0 (reference)	1.0 (reference)
Current use of low-dose aspirin and a PPI: >1 month PPI duration	209 (21.2)	458 (21.2)	0.79 (0.63–1.00)	0.69 (0.54–0.88)
Primary CVD prevention	96 (9.7)	252 (11.7)	0.68 (0.51–0.91)	0.61 (0.45–0.83)
Secondary CVD prevention	113 (11.4)	206 (9.5)	0.92 (0.69–1.23)	0.79 (0.58–1.06)

*Adjusted by matching variables (sex, age and calendar year) and number of PCP visits in the year before the index date.

†Adjusted by matching variables (sex, age and calendar year) and number of PCP visits in the year before the index date, alcohol consumption, smoking, history of UGIB, LGIB, GIB, peptic ulcer, pancreatic disease, polytherapy and use of clopidogrel, NSAIDs and warfarin.

CI, confidence interval; CVD, cardiovascular disease; GIB, gastrointestinal bleeding; LGIB, lower gastrointestinal bleeding; NSAID, non-steroidal anti-inflammatory drug; OR, odds ratio; PCP, primary care practitioner; PPI, proton pump inhibitor; UGIB, upper gastrointestinal bleeding

Supplementary Table 3. ORs (95% CIs) for the association between concomitant use of DAT and a PPI and the risk of UGIB.

	UGIB N=987 n (%)	Controls N=2160 n (%)	Crude OR (95% CI)	Adjusted OR (95% CI) *
Current use of DAT and past use of a PPI (**reference group**)	15 (1.5)	14 (0.6)	1.0 (reference)	1.0 (reference)
Current use of DAT and current use of a PPI	36 (3.6)	29 (1.3)	1.16 (0.48–2.79)	0.62 (0.31–1.25)
Current use of DAT and a PPI: ≤1 month PPI duration	8 (0.8)	3 (0.1)	2.49 (0.55–11.31)	0.89 (0.15–5.49)
Current use of DAT and a PPI: >1 month PPI duration	28 (2.8)	26 (1.2)	1.01 (0.41–2.48)	0.61 (0.30–1.23)
Current use of DAT and never use of a PPI	38 (3.9)	29 (1.3)	1.22 (0.51–2.93)	0.86 (0.41–1.80)

*Adjusted by matching variables (sex, age and calendar year) and number of PCP visits in the year before the index date, alcohol consumption, smoking, history of UGIB, LGIB, GIB, peptic ulcer, pancreatic disease, polytherapy and use of clopidogrel, NSAIDs and warfarin.

CI, confidence interval; DAT, dual antiplatelet therapy; GIB, gastrointestinal bleeding; LGIB, lower gastrointestinal bleeding; NSAID, non-steroidal anti-inflammatory drug; OR, odds ratio; PPI, proton pump inhibitor; UGIB, upper gastrointestinal bleeding

Supplementary Table 4. ORs (95% CIs) for the association between concomitant use of DAT and a PPI and the risk of LGIB.

	**LGIB N=1428 n (%)**	**Controls N=4100 n (%)**	**Crude OR (95% CI)**	**Adjusted OR (95% CI)***
Current use of DAT and past use of a PPI (**reference group**)	26 (1.8)	29 (0.7)	1.0 (reference)	1.0 (reference)
Current use of DAT and current use of a PPI	37 (2.6)	56 (1.4)	0.74 (0.38–1.44)	0.97 (0.39–2.42)
Current use of DAT and a PPI: ≤1 month PPI duration	3 (0.2)	3 (0.1)	1.12 (0.21–6.02)	1.96 (0.41–9.41)
Current use of DAT and a PPI: >1 month PPI duration	34 (2.4)	53 (1.3)	0.72 (0.36–1.42)	0.85 (0.33–2.18)
Current use of DAT and never use of a PPI	28 (2.0)	47 (1.1)	0.66 (0.33–1.35)	1.35 (0.54–3.35)

*Adjusted by matching variables (sex, age and calendar year) and number of PCP visits in the year before the index date, alcohol consumption, smoking, BMI, history of polyps, LGIB, unspecified GIB, peptic ulcer, GERD, IBD, IBS, polytherapy and use of clopidogrel, NSAIDs and warfarin.
BMI, body mass index; CI, confidence interval; DAT, dual antiplatelet therapy; GERD, gastro-esophageal reflux disease; GIB, gastrointestinal bleeding; IBD, inflammatory bowel disease; IBS, irritable bowel syndrome; LGIB, lower gastrointestinal bleeding; NSAID, non-steroidal anti-inflammatory drug; OR, odds ratio; PCP, primary care practitioner; PPI, proton pump inhibitor
